# On re-defining public health and making health public

**DOI:** 10.1093/pubmed/fdaf095

**Published:** 2025-12-10

**Authors:** John Coggon

**Affiliations:** Centre for Health, Law, and Society, University of Bristol, Bristol, UK

The public health responsibilities of governments and other organizations are built on philosophical foundations.^1^ Practically, these are rooted in a focus on health at a population level, and the protection and promotion of health through shared political and societal activities.^2^ From ‘within’ public health, it is almost trite to link those foundations with two specific moral missions: first, to protect and promote good health as an imperative that may, and sometimes must, overrule competing values and preferences; second, an imperative to address unfair, systemically-determined health inequalities.^3^ Importantly, whilst these imperatives may be almost taken-for-granted mantras of ethical public health as a vocational practice, the philosophical bases of public health responsibilities are grounded too—perhaps predominantly—in the ideological commitments and priorities of sitting governments and the legislators that empower and constrain public health activity. This has implications sub-nationally, nationally, and supranationally for the applied meaning of public health.

In the abstract, public health has at its heart the reasons why, and the extent to which, health is a shared concern that is to be addressed through shared practices and institutions.^4^ As something to be observed and described, public health is defined as much by what is (not) done in its name as it is by how it is characterized in different statements by august organizations or specialist textbooks.^5^ And given its practical grounding, disagreements on the proper meaning and scope of public health aims and agendas are political disagreements. A key aspect of public health leadership is thus advocacy for the values and knowledge bases that underpin shared health goals—including in instances where this may come into tension with national and transnational health priorities as cast by political leaders.^6^ Such advocacy requires public health leaders to articulate their vision of public health, of what gives it legitimacy, and what this means for the creation of different methods of public health intervention.^7^

Political and ideological differences, and discussion of advocacy, inevitably give rise to questions of friction in the idea of making health public.^8^ It is possible and productive to explore such advocacy through a lens of ‘us against them’: for instance, by reference to battles about the commercial determinants of ill health and health inequalities, including as these feed into domains of political decision-making.^9^ However, there is important scope too in exploring the meaning of public health, and the practices of making health public, through critical self-reflection; through analysis of debates amongst protagonists who share the broad philosophical commitments within public health, but who differ on their detail.

Based on a scoping review, it has recently been argued that public health has not been re-defined for about 20 years and that this is problematic because it suggests a failure to keep pace with new and changing challenges.^10^ Yet alternative approaches to addressing the question of whether we find definition and re-definition show that public health leaders, researchers, and practitioners have been advancing redefinitions: and they have been doing so precisely because of changing dynamics, situations, and understandings. Most starkly, these redefinitions have come in characterizations of, and advocacy for, alternative framings to bring emphasis to important and missed (or inadequately appreciated) public health concerns. Without aiming to be comprehensive, we might first list here the conceptual and practical rise of the idea of global health,^11^ and more recently of Planetary Health, One Health, and EcoHealth.^12^ Each of these is both a philosophical critique of the proper orientations, reach, and priorities of public health, and a practical call to give effect to proper points of focus of public health activity.

That pitching of alternative terms to capture meanings of public health has run in notable parallel with growing engagement with questions about governance; about the depth and detail, to take Charles-Edward A. Winslow’s celebrated phrase, of ‘organized community efforts’.^13^ This focus has been on manifestations of governance for and against shared health interests, with an understanding that key governance actors exist beyond governments, international organizations, and public agencies and authorities.^14^ This in turn presents challenges about roles and remits beyond serving the public interest. And it presents challenges about the applicability (or otherwise) of institutional values such as transparency, scrutiny, and accountability. And it invites careful scrutiny of the role and authority of legal and other methods of regulation.^15^

Where, positively, do the above reflections bring us if we want to understand what public health is and what it should be, and to translate philosophy into action? First, we find a firm reminder of the need for historical and social contextualization. Public health ‘happens’. It happens in contexts. And those contexts themselves place limits on what might be possible. Awareness is needed of limitations and possibilities if ambitions to make health public in particular ways are to be realized. Secondly, we must not forget that as well as conceptual discourses on the meaning of public health, there are vast resources within the well-established fields of critical public health and the philosophy of public health. These bring meanings and rigour to public health, for instance, in critically identifying which health inequalities matter and why,^16^ or in establishing conceptual, motivational, methodological, epistemological, evaluative, and practical concepts that shape and might shape public health imperatives.^17^

I would argue that the defining and re-defining of public health is a task that is achieved through critical debate and the advancing and defending of positive visions for practices that would make health public. The focal point is health and health-affecting phenomena, with health recognised as a shared value that is the proper target of shared societal efforts. In approaching this, we need to consider outright threats to health. But we also need to look past headline commitments, for instance, to social justice, and drill into what a commitment to justice looks like and why. This means exploring points of subtlety, distinctions, and disagreements in what we might identify as unfair social arrangements, opportunities, and outcomes, and where and why we draw the line on permissible versus impermissible methods of intervention.

In relation to public health aims, we might ask, for example, whether a commitment to a planetary health approach has at its base a concern for planetary environments and ecosystems generally, or for these insofar as their protection is conducive to sound conditions for the human species. Or we might question the meaning and reach of ‘global’ in ideas framed as global health, and ask whether its limits belie rather than support claims made in its name. In relation to the means of making health public, we might ask if it is right, for instance, that we fixate on—and implicitly endorse—received wisdoms on the neutrality of ‘nudges’ as regulatory tools, whilst simultaneously seeking high justificatory demands in legal regulations that might effect public health aims. Or we might ask if addressing instances of specific health inequity, whilst failing to address the underpinning—upstream—socio-economic context that leads to that inequity, actually helps perpetuate rather than address social injustice.

Such challenges need to be raised and talked through in constructive dialogues. The questions may be unsettling, but asking them allows a clearer understanding of values-based claims and where commitments are shared, qualified, or after all reflective of points of profound disagreement. At the heart of public health research, practice, and policy is the general aim to make health public. In its particulars, however, the value judgements—the questions of ethics and equity—are far from straightforward. Acknowledgment of that is not a failing. Rather, engagement with it can turn platitudes into meaningful principle, and underpin advocacy that is attuned to the complexities of the contexts that good health policy and practice have a key role in addressing.

## Notes

**John Coggon,** Professor of Law
